# Improving debriefing practices for participants in social science experiments

**DOI:** 10.1093/pnasnexus/pgae502

**Published:** 2024-12-03

**Authors:** Katherine Clayton, Ethan Porter, Yamil Velez, Thomas J Wood

**Affiliations:** Department of Sociology, Stanford University, Building 120, 450 Serra Mall, Stanford, CA 94305, USA; Department of Political Science, George Washington University, 21st St NW, Washington, DC 20052, USA; Department of Political Science, Columbia University, 420 W 118th St, New York, NY 10027, USA; Department of Political Science, Ohio State University, 154 North Oval Mall, Columbus, OH 43210, USA

**Keywords:** social science experiments, misinformation, research design, research ethics

## Abstract

Social science experiments often expose participants to false, deceptive, or otherwise harmful content. In an effort to mitigate the effects of such content and to comply with regulatory standards, these studies usually conclude by “debriefing” participants about the content they encountered, on the assumption that doing so will eliminate the effects of exposure. We present evidence showing that this assumption is not always correct. After standard debriefs, participants who have seen political misinformation often remain worse-informed than if they had never been exposed to misinformation in the first place. We then design and test a new approach to debriefing, which entails (i) debriefing as soon after exposure to harmful content as possible; (ii) providing an informative correction; and (iii) requiring participants to affirm that they have been exposed to false information. Across multiple experiments, we show that this approach is far superior to standard debriefs at reducing the effects of false or harmful content on political attitudes and beliefs. Our approach makes it possible to study the effects of false or harmful content on attitudes and beliefs without posing significant risks to participants.

## Introduction

Experiments in the social sciences that involve exposing participants to false, misleading, or dangerous information often conclude by “debriefing” participants about the content they have seen and/or the purposes of the research study. In the United States, debriefing statements are required by Institutional Review Boards (e.g. (1–4)) and may be delivered on the assumption that they can “undo” the effects of exposure to such content. Social scientists use potentially harmful stimuli to study a wide range of topics, including the effects of misinformation on attitudes about election legitimacy or vaccination (5, 6), the relationship between political rhetoric and violence (7), the effects of norm-eroding statements on respect for democratic norms (8), and whether descriptions of partisan conflict make voters more likely to dehumanize members of the opposing party (9). These processes are vital to understand, yet studying them through experiments that randomize exposure to harmful content risks leaving participants worse off than they were before entering the study.

Despite the prevalence of debriefs in the social sciences, evidence on their effectiveness is scant. Though debriefs may satisfy researchers’ obligations to participants in the eyes of Institutional Review Boards, we know little about their impact on participants’ attitudes and beliefs, including whether debriefs remediate the harms of the content which necessitated the debrief in the first place. It is possible that debriefs achieve their objectives; it is also possible that they fail, leaving study participants harmed in their wake. Differently designed debriefs, however, may prove more effective.

In this paper, we measure the effectiveness of “standard” debriefs (i.e. those required by most university IRBs and in wide use today) and present evidence in favor of a more effective approach. We do so in the context of misinformation. Not only is misinformation an urgent issue that receives considerable attention from scholars and policymakers (10, 11), but also the design of many misinformation experiments—presenting participants with harmful content and then debriefing at the end of the study—lends itself well to the study of debriefs.

We begin by assessing whether exposure to standard debriefs negates the effects of exposure to misinformation. We do so by replicating existing misinformation research and randomizing whether the debrief occurs before or after measures of belief in false statements. In this initial set of studies, standard debriefs fail to achieve their objective. Participants in political misinformation experiments remain affected by misinformation, despite being exposed to a standard debrief.

We then design and test a new approach to debriefing—one that (i) is presented to participants as early as possible in a survey, (ii) implements a fact-check style correction to the misinformation, and (iii) compels participants to actively acknowledge that they were exposed to false content during the study. This new and improved debrief stands in stark contrast to standard debriefs, which tend to occur at the very end of an online survey, are often vague in terms of their corrective content, and present participants with debriefing information passively. Across multiple experiments, we find that our improved debrief bests the standard debrief at ameliorating the effects of harmful content, while leaving treated participants better informed than they would have been otherwise.

## The limited effectiveness of standard debriefs

To diagnose the problem, we first replicate well-known studies focused on misinformation related to COVID-19 (6) and elections (5). Participants (n=1,827) were recruited over Lucid and randomly assigned to conditions that (i) presented misinformation, collected outcome measures, then debriefed, as is standard practice; (ii) presented misinformation, then debriefed, then collected outcome measures; or (iii) presented placebo content, then collected outcome measures (with no exposure to misinformation). For both studies, outcome measures gauged belief in the underlying misinformation (6) and also looked at attitudes and belief intentions related to COVID-19 vaccines.

Results reveal that debriefs do not effectively reduce belief in politically charged misinformation. For instance, vaccine hesitancy increased by 14.3 percentage points (SE=0.036) relative to the placebo content directly after exposure to the misinformation treatment, but it also increased 8.4 percentage points (SE=0.029) relative to the placebo when respondents were debriefed before the outcome measures were collected. The difference between these two conditions (5.9 percentage points, SE=0.036) was not statistically significant, suggesting that participants retained significant belief in misinformation after the standard debriefing procedure was deployed. For the election misinformation study, we failed to replicate misinformation effects in the full sample, but observed debriefs failing across several outcome specifications among Republicans, who tend to be less trusting of elections in the contemporary political climate at baseline (8).

In an additional study, we used the same design described above on Amazon Mechanical Turk respondents (n=1,739) to test the effectiveness of debriefs in the context of nonpolitical myths, such as the idea that toilets flush in opposite directions in each hemisphere. In these examples, debriefs are more consistently effective, with all point estimates running in a negative direction (implying more accurate beliefs after exposure). Standard debriefs work effectively when they target innocuous issues that participants are unlikely to have strong prior beliefs about. But when standard debriefs target political misinformation, of the sort that many scholars are interested in, they often fail.

## Designing effective debriefs

**Fig. 1.**
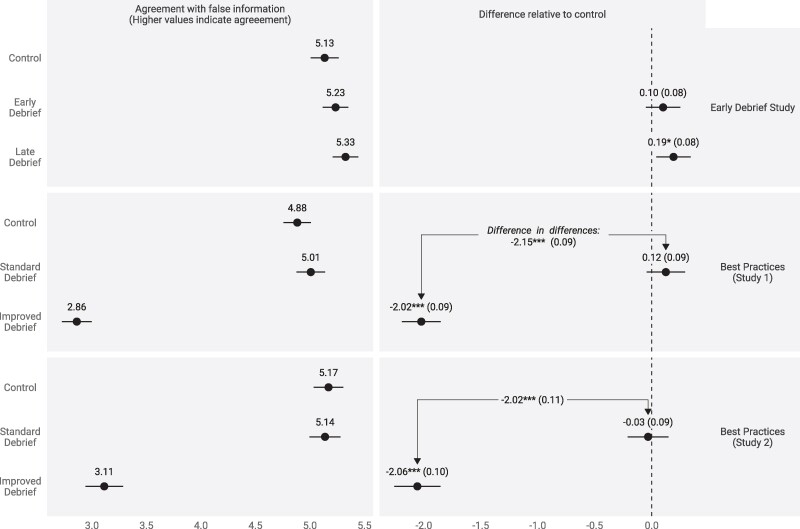
Studies on improving debrief effectiveness.

Observing that debriefs fail in the context of political misinformation, we devised and tested debriefs meant to improve upon current practices (see Materials and methods for more details on study design). In the Early Debrief Study (Figure 1, top row; *P < .05, **P < .01, ***P < .001), we study debriefing participants as quickly as possible, immediately after outcome measurement; we find this approach worked only for one partisan group (Democrats) and was ultimately ineffective among the full sample.

Our Best Practices studies (second and third row of Figure 1) offer evidence in support of a debriefing technique that consistently succeeds relative to a standard debrief or a no-information control condition. Participants randomly assigned to the best practices debrief:

1. saw an extensive debrief (in the form of a fact-check) immediately after outcome measurement;
2. and were required to acknowledge, via a checkbox, that the content they saw was inaccurate and that they were exposed to it only for a research project.

The first of these two studies (Best Practices Study 1) (n=2,789) was conducted on CloudResearch Connect; Best Practices Study 2 (n=1,983) was a replication of Best Practices Study 1, conducted on Lucid. Consult the Materials and methods for more details about the effective debrief. As the bottom two rows of Figure 1 illustrate, in both studies, the improved debrief improved belief accuracy by more than two points on a seven-point scale, or an effect size of 1.25. The standard debrief had no discernible effect.

## Discussion

Standard debriefs that follow the bare minimum requirements of university IRBs are not always effective. Our results show that they are especially likely to fail when they target partisan content for which participants hold stronger beliefs about (12)—the precise beliefs that social scientists may be most interested in studying. Even with IRB approval, these debriefs may fall short of fully addressing the Belmont Report’s beneficence principle (13). Insofar as they do not eliminate the effects of harmful content on beliefs, they leave participants worse off, and thus more harmed by their participation, than they would be otherwise.

Fortunately, by exposing people to comprehensive corrections and then requiring them to affirm the falseness of the content to which they were exposed, we are able to bring participants back to baseline, thereby meeting the standards of the beneficence principle. Future research should investigate the extent to which participants in the Best Practices debrief sincerely and/or durably change their beliefs, perhaps with a longitudinal design. Future research should also study whether any design modifications can make the Best Practices debrief even more effective and/or efficient. Indeed, there may be other ways to effectively debrief that are less time-intensive, such as incorporating just one or two elements of our compound Best Practices treatment. Finally, we welcome research on whether our debrief remains effective across a wider range of political contexts and topics.

Broadly, our results should challenge scholars’ assumptions about the efficacy of the debriefs they use and lead to better approaches.

## Materials and methods

Participants in all of our studies were recruited from online sample providers regularly used for research in the social sciences (e.g. (14, 15)) including Lucid Theorem (COVID/Election Misinformation Replications, Best Practices Study 2), Amazon Mechanical Turk (Apolitical Debriefs Study), and Cloud Research (Early Debrief Study, Best Practices Study 1). We implemented two attention checks prior to treatment and omitted those who failed. Samples recruited from Lucid Theorem are diverse and approximate the adult population in the United States based on age, gender, ethnicity, and region. For samples recruited from CloudResearch, we targeted approximately 50% Democrats and 50% Republicans, as experimental stimuli differed depending on respondents’ partisan affiliation.

All studies were preregistered at the Open Science Framework prior to data collection (COVID/Election Misinformation Replications: https://osf.io/awtfd; Apolitical Debriefs Study: https://osf.io/2sxba; Early Debrief Study: https://osf.io/ft73g; Best Practices Study 1: https://osf.io/5gksj; Best Practices Study 2: https://osf.io/zey4n), programmed in Qualtrics, and fielded online. Fielding dates are as follows: COVID/Election Misinformation Replications (3/28/22-4/14/22); Apolitical Debriefs Study (6/22/22-7/4/22); Early Debrief Study (10/28/22-11/08/22); Best Practices Study 1 (5/22/23-6/16/23); Best Practices Study 2 (7/12/23-7/14/23). The studies received approval from the Office of Human Research at the George Washington University Institutional Review Board (Protocol NCR224025). Informed consent was obtained from all study participants. Median response times ranged from 2 minutes, 37 seconds (Apolitical Debriefs Study) to 6 minutes, 18 seconds (COVID/Election Misinformation Replication).

Participants in the Best Practices Studies assigned to the effective debrief condition were first exposed to a comprehensive correction of the misinformation they had seen. They were then presented with text, the first sentence of which read: “By clicking this button, I affirm that I know I was exposed to false information for the purpose of this research.” The second sentence varied based on the false content they had seen. For example, those assigned to read misinformation and then be debriefed about COVID-19’s origins saw: “There is no evidence that COVID-19 was created in a Chinese lab.” Below this text, a button appeared, with the text “I affirm” adjacent to the button.

For each study, we analyze our data using OLS regression with HC2 robust standard errors. We regress each outcome on an indicator for treatment condition. Where appropriate, we conduct analyses among Democrat and Republican subgroups (e.g. (5) replication in COVID/Election Misinformation Replications; party-specific misinformation stimuli used in Early Debrief Study and Best Practices Studies) in addition to running our main models. Our significance threshold is P<0.05.

## Data Availability

Data and code necessary to replicate the findings in this article are available at the Open Science Framework (https://osf.io/pu8mw/).
